# Proteomic analysis of regenerated rabbit lenses reveal crystallin expression characteristic of adult rabbits

**Published:** 2008-12-19

**Authors:** Xialin Liu, Min Zhang, Yuhua Liu, Pratap Challa, Pedro Gonzalez, Yizhi Liu

**Affiliations:** 1 Zhongshan Ophthalmic Center, State Key Laboratory of Ophthalmology, Sun Yat-sen University, Guangzhou, China; 2 Department of Ophthalmology, Duke University, Durham, NC

## Abstract

**Purpose:**

To explore lens crystallin characteristics and morphology of rabbit regenerated lenses in comparison with wild type natural lenses by means of proteomic analysis and histological assay.

**Methods:**

The lens regeneration model of the New Zealand rabbit was established, and lens regeneration was observed by slit lamp examination and photography. A histological assay was evaluated under light microscopy and transmission electron microscopy (TEM). Protein samples of regenerated lenses were collected from experimental rabbit eyes 2, 4, and 16 weeks after surgery. Two-dimensional gel electrophoresis (2-DE) was performed. Image analyses was done using the ImageMaster 2D Elite 3.01 software package. The protein spots were trypsinized and identified by matrix-assisted laser desorption/ionization-time-of-flight-mass spectrometry.

**Results:**

Lens regeneration began in the periphery of the capsule bag about one to two weeks after the surgery and proceeded to regenerate toward the center. The regenerated lens appeared spherical in shape with a fairly translucent cortical structure and a nuclear opacity. Histological findings showed that the remnant lens epithelial cells differentiate at the lens capsule equator and new lens fibers form in a concentric pattern in a manner similar to that observed in natural lenses. However, TEM showed morphological changes in the epithelial cells of the regenerated lenses as compared with natural lenses. 2-D electrophoresis revealed that the patterns of protein spots from regenerated lenses (two weeks, four weeks, and 16 weeks) were analogous to those of 16-week-old natural lenses but were substantially different from those of two-week-old natural lenses, particularly when the two-week-old regenerated lenses were compared with the two-week-old natural lenses.

**Conclusions:**

Proteomic analysis revealed that crystallin expression in regenerated rabbit lenses was analogous to that of natural lenses of adult rabbits but was different from that of very young rabbits (two weeks old), and TEM revealed the presence of morphological changes in the epithelial cells of regenerated lenses. These results suggest that the regrowth of lens materials in the lens capsule after endocapsular phacoemulsification might actually represent the regeneration of “mature” lens substances, which have led us to the conclusion that the regenerative process does not exactly mimic embryonic development.

## Introduction

Cataract phacoemulsification surgery and intraocular lens implantation is highly successful. However, it is associated with accommodation dysfunction and various complications [1]. Since 1827 [2], spontaneous regeneration of the lens following extracapsular extraction has been extensively studied in rabbits as well as other mammals [3-9]. Reports of cell regrowth in animal lenses led us to consider whether regeneration of human lenses might eventually be possible [10]. Therefore, we have undertaken studies to further understand the process of regeneration in the rabbit lens.

It has been demonstrated that after lens substance evacuation, the remnant lens epithelial cells differentiate at the lens capsule equator and new lens fibers form first in the equatorial region. They then align with each other in a concentric pattern in a manner similar to that observed in embryonic and young rabbits [11].The newly formed lens contains all of the major crystallin classes, although several specific crystallin subunits have been found to be absent or present in abnormally low concentrations [12]. Embryonically, lens development involves a process of continuous proliferation and differentiation of lens epithelial cells. As in embryonic development, regeneration of rabbit lens proceeds by cellular proliferation and differentiation along the capsule [13,14]. It has been hypothesized that lens epithelial cells at the equatorial zone may have features of stem cells, i.e. the ability to proliferate and differentiate into lens fibers and finally form a completely regenerated lens [8,15-18]. Recent studies, however, have reported that even stem cells are not exempt from aging [19-22]. Therefore, we are interested in exploring whether adult lens epithelial cells can really regenerate lens substance by mimicking the process of the lens development.

Given the similarity between the processes of development and regeneration of rabbit lens [3,5,6,13], we have studied the histological features and the profile of the protein composition of regenerated lens materials and compared them with the natural lens materials from rabbits of different ages. A perfect regenerated lens should have the healthy appearance and histological arrangement of a new regenerated lens as well as an accurate protein composition. In this study, we established a lens regeneration model in the New Zealand rabbit as previously  reported [14,23], observed the process of regeneration and the histological features, and performed proteomic analysis to explore the characteristics of the lens proteins.

## Methods

### Establishment of regenerated rabbit lens model

All the experimental protocols using animals strictly adhered to the ARVO Statement for the Use of Animals in Ophthalmic and Vision Research under an approved animal protocol. Following Gwon’s method, lens extraction was performed by endocapsular phacoemulsification on the experimental eyes of 12-week-old New Zealand albino rabbits (weighing 1.5–2.5 kg) [14,23]. Following lens extraction, all treated eyes received topical 1% tropicamide and 0.3% tobramycin four times daily for seven days. Lens regeneration was evaluated by slit lamp examination, and photographs were taken. Rabbits were divided into groups of three, which were then sacrificed at different times (two weeks, four weeks, and 16 weeks). The regenerated lens from each rabbit was dissected carefully and stored under different conditions, depending upon the analysis techniques to be used. Controls were natural lenses from 2-, 4-, and 16-week-old wild-type rabbits.

### Sample preparation for histopathological assay

#### Paraffin-embedded tissue section preparation

The whole, dissociated, regenerated lenses and natural control lenses were fixed in 10% neutral buffered formalin. Tissue was processed in an automatic tissue processor overnight and was dehydrated in reagent grade alcohol, cleared with xylene, and infiltrated with paraffin. The paraffin embedded tissue was sectioned at 5 µm and stained with hematoxylin and eosin.

#### Tissue preparation for transmission electron microscopy

Lens capsules were removed from the lens cortex. The capsule specimens were immediately placed in a 1% glutaraldehyde/4% formaldehyde solution and sent to the pathology laboratory. Post-fixation was performed in osmium tetroxide. The capsule specimens were dehydrated in increasing concentrations of ethyl alcohol and embedded in resin. Ultrathin, 100 nm sections were stained with uranyl acetate and lead citrate, and transmission electron microscopy (TEM) was performed with a Philip CM-10 electron microscope (Philip, Eindhoven, The Netherlands). The TEM slides were reviewed in a masked fashion.

### Sample preparation for protein analysis

Lens samples were collected from experimental regenerated lenses at different times (2, 4, and 16 weeks after surgery) and from natural control lenses. The capsules of the lenses were removed, and the lens materials were pooled and ground to fine powder with a mortar in the presence of liquid nitrogen. The powder was then dissolved in lysis buffer that contained 7 M urea, 2 M thiourea, 2% w/v 3-[(3-Cholamidopropyl)dimethylammonio]propanesulfonic acid (CHAPS), 1% dithiothreitol (DTT), 2%V/V carrier ampholyte, pH 3–10, and protease inhibitor cocktail mix (Boehringer Mannheim GmbH, Mannheim, Germany) and then spun at 12,000 rpm at 4 °C for 20 min. The supernatant was collected and stored at −70 °C until further use. Protein concentration was measured by the Bradford assay [24].

### Two-dimensional electrophoresis

The proteins of all samples were first characterized using 13% SDS–PAGE. The concentration of the sample was 2 mg/ml, and the amount was 20 μl/band. The gels were stained with Coomassie brilliant blue R-250 (Sigma-Aldrich Corp., St. Louis, MO). Two-dimensional gel electrophoresis (2-DE) was performed as described by Gorg [25] using precast immobilized pH gradient (IPG) strips (immobiline DryStrip pH3-10 NL,18cm; GE Healthcare Life Science, Piscataway, NJ) in the first dimension (isoelectric focusing) according to the manufacture’s instructions, and SDS–PAGE in the second dimension. Total protein (150 μg) was loaded on each IPG strip. After separation, the strips were immediately equilibrated two times for 15 min each time; in the first 15 min, the strips were equilibrated with 50 mM Tris-HCl, pH 8.8, 6 M urea, 30% glycerol, 2% SDS, and DTT (0.5% W/V). In the second 15 min step, 4.5% w/v iodoacetamide, but not DTT was added to the equilibration solution.to alkylate thiols. The separation in the second dimension was performed using 13% SDS–PAGE gel in the Protein II device (Bio-Rad, Hercules, CA). The strips were held in place with 0.5% agarose dissolved in SDS/Tris/glycine containing running buffer, and electrophoresis was performed at a constant current (30 mA/gel) at 16 °C. After electrophoresis, gels were stained with Coomassie brilliant blue R-250.

### Image analysis

The stained 2-D gels were imaged using the ImageScanner (Amersham Pharmacia Biotech). Images were digitized and evaluated with ImageMaster 2D Elite 3.01 software (Amersham Pharmacia Biotech). Image analysis was conducted for spot detection, matching, background subtraction, normalization, and isoelectric point/molecular weight calibration.

### Mass spectrometry

Trypsin in-gel digestion was performed as described by Rosenfeld et al. [26]. Briefly, gel spots were excised from the stained gel and cut into 1–2 mm^2^ slices then destained with 25 mM ammonium bicarbonate/50% acetonitrile (Fisher Scientific, Springfield, NJ) and lyophilized with a SpeedVac Plus SC110A vacuum concentrator (Savant, Holbook, NY). The gel was rehydrated in trypsin solution (Boehringer Mannheim). The ratio of enzyme to protein was about 1:20 [27]. After overnight incubation at 37 °C, peptides were eluted with 5% trifluoroacetic acid (TFA) at 40 °C for 1 h followed by 5% TFA/50% acetonitrile elution until the gel slices became white. The eluate was collected in an Eppendorf tube and lyophilized with SpeedVac Plus SC110A. The peptide mixture was dissolved with 0.5% TFA for mass spectrometry analysis. The peptide mixture with matrix solution, α-cyano-4-hydroxycinnamic acid (CHCA; Sigma, St. Louis, MO), was measured on matrix-assisted laser desorption/ionization-time-of-flight-mass spectrometry (MALDI-TOF-MS [Reflex III; Bruker, Billerica, MA]) fitted with N_2_ lasers. The protein search was performed on the Matrix Science  website with the search parameters set as follows: enzyme: trypsin, mass values: monoisotopic, Peptide Mass Tolerance: ±0.5 Da, Peptide Charge State: 1+, Max Missed Cleavages: 1.

## Results

### Shape and transparency of regenerated lens

**Figure 1:**
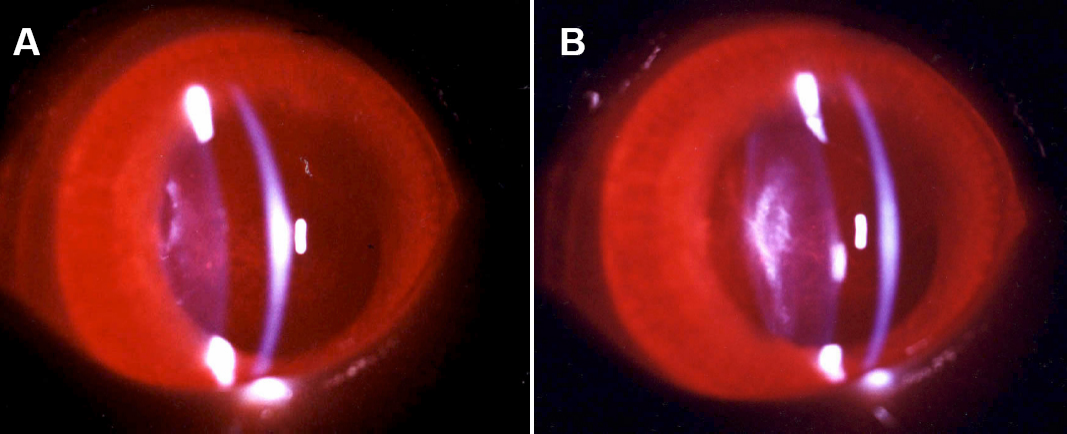
Representative regenerated lens at 12 weeks observed by slit lamp examination and photography. **A**: Relatively clear lens substance is seen in the periphery. **B**: Central opacity is visible. The capsular bag was almost completely filled with a new regenerated lens by the end of 12−16 weeks.

**Figure 2:**
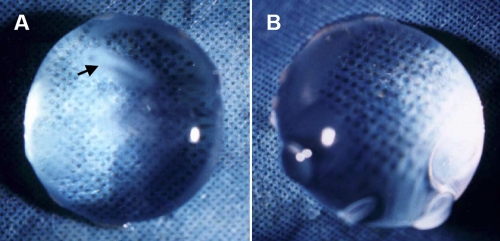
Regenerated lens at 16 weeks was compared with natural control lens. **A**: The representative photograph shows the fully regenerated lens, which looked flatter than the natural lens and quite translucent. It has a star-shaped nuclear opacity and some opaque spots. The anterior capsulotomy site were indicated by a small black arrow. **B**: A natural control lens from a 16-week-old rabbit is shown.

During the first one to two weeks after the surgery, lenses regenerated along the periphery of the capsular bag between the anterior and posterior capsules. With time, the earliest regenerated lens fibers became progressively compacted and pushed toward the center of the capsular bag. Finally, progressive regrowth resulted in a regenerated lens exhibiting star-shaped nuclear opacity. Cortical lens fibers, which were produced in the later stages of regeneration, appeared quite translucent. The capsular bag was almost completely filled with a new, regenerated lens by the end of 12-16 weeks (Figure 1). After the fully regenerated lens (16 weeks) was removed from the eye, it appeared spherical in shape (less round than a normal lens) and had a fairly translucent cortical structure with some opaque spots and a star-shaped nuclear opacity (Figure 2).

### Histological findings

**Figure 3:**
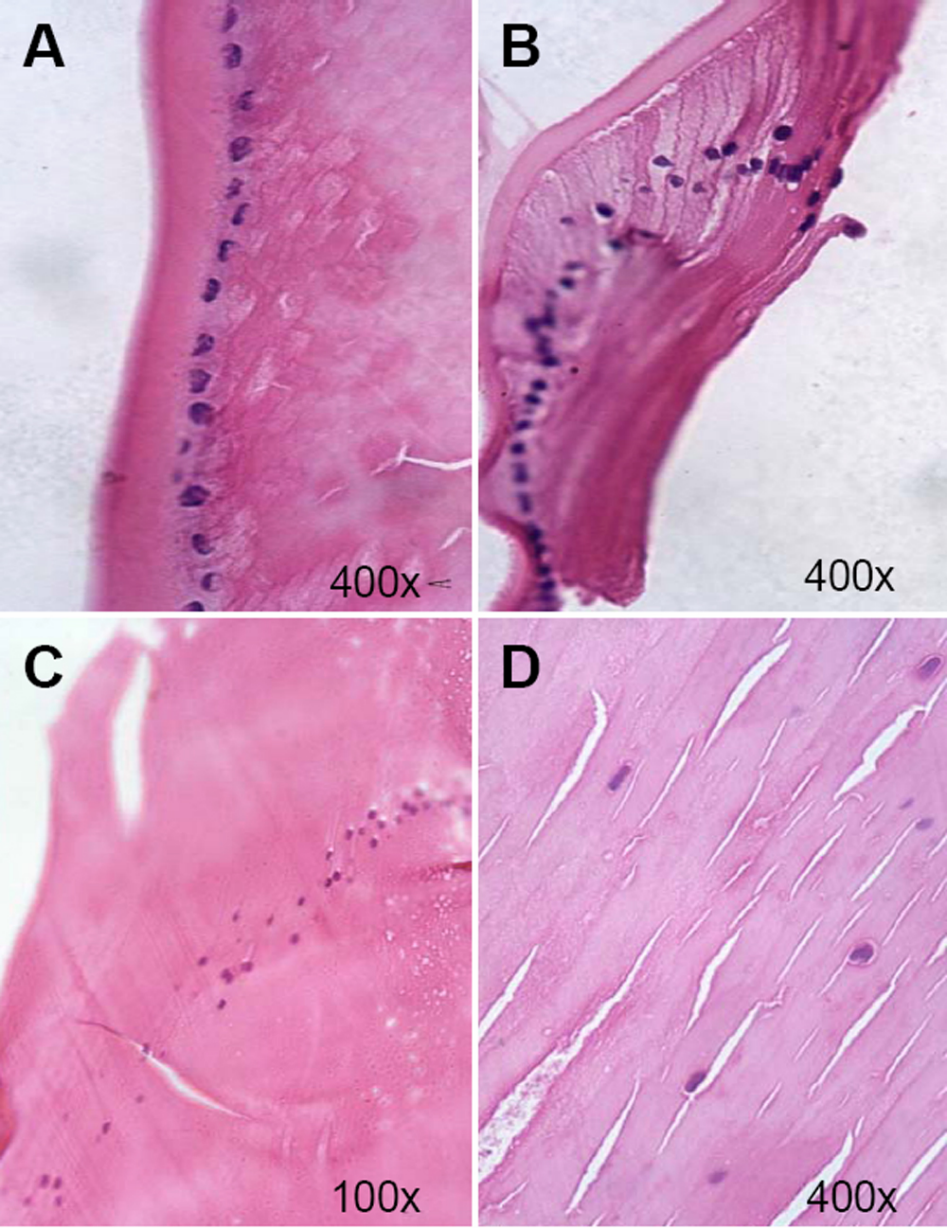
Histological characteristics of regenerated lens seen by light microscopy. **A**: A single layer of the lens epithelial cell lines the anterior capsule. Cells are similar to epithelial cubical cells morphologically. **B**: Partial cell differentiation is seen in the equatorial zone. **C**: Proliferated epithelial cells have elongated in an anterior-posterior direction, forming a classical “arch zoster” due to anterior displacement of the nuclei. **D**: Lens fiber alignment was uniformly arranged in the regenerated lens, particularly at the periphery.

**Figure 4:**
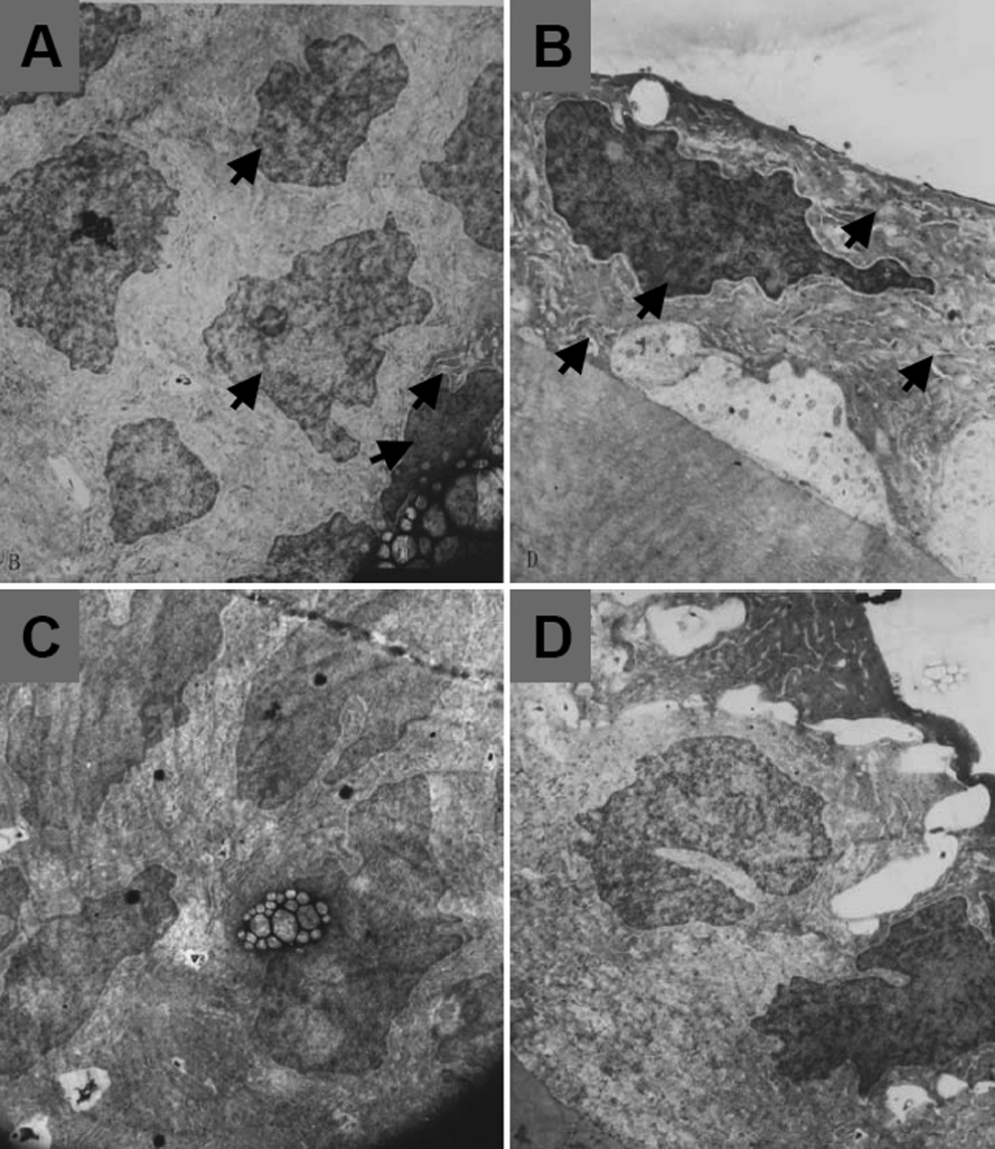
Representative transmission electron micrograph of epithelial cells in a regenerated lens. Morphological changes are seen both at the peripheral equatorial (**A**) and central (**B**) areas, including overly dense and indented nuclei, edematous mitochondria, and an expanding endoplasmic reticulum when compared to natural lenses (**C**,**D**). Magnification is****8,000X).

Light microscopy revealed a single layer of lens epithelial cells lining the anterior capsule (Figure 3). The nearer the cells were to the equator, the more similar they were to epithelial cubical cells morphologically. Partial cell differentiation was seen in the equatorial zone. As in natural lenses, lens epithelial cells in regenerated lenses proliferate and subsequently elongate in an anterior-posterior direction, forming a classical “arch zoster” due to anterior displacement of the nuclei. A similar cellular morphological change was noted in lens fiber differentiation. Lens fiber alignment was uniform in the regenerated lens, particularly in the peripheral area. However, TEM (Figure 4) revealed that compared with natural lenses, the epithelial cells of the regenerated lenses had some morphological changes in both the central and peripheral equatorial areas, showing overly dense, indented nuclei, some edematous mitochondria, and an expanding endoplasmic reticulum.

### Protein analysis

**Figure 5:**
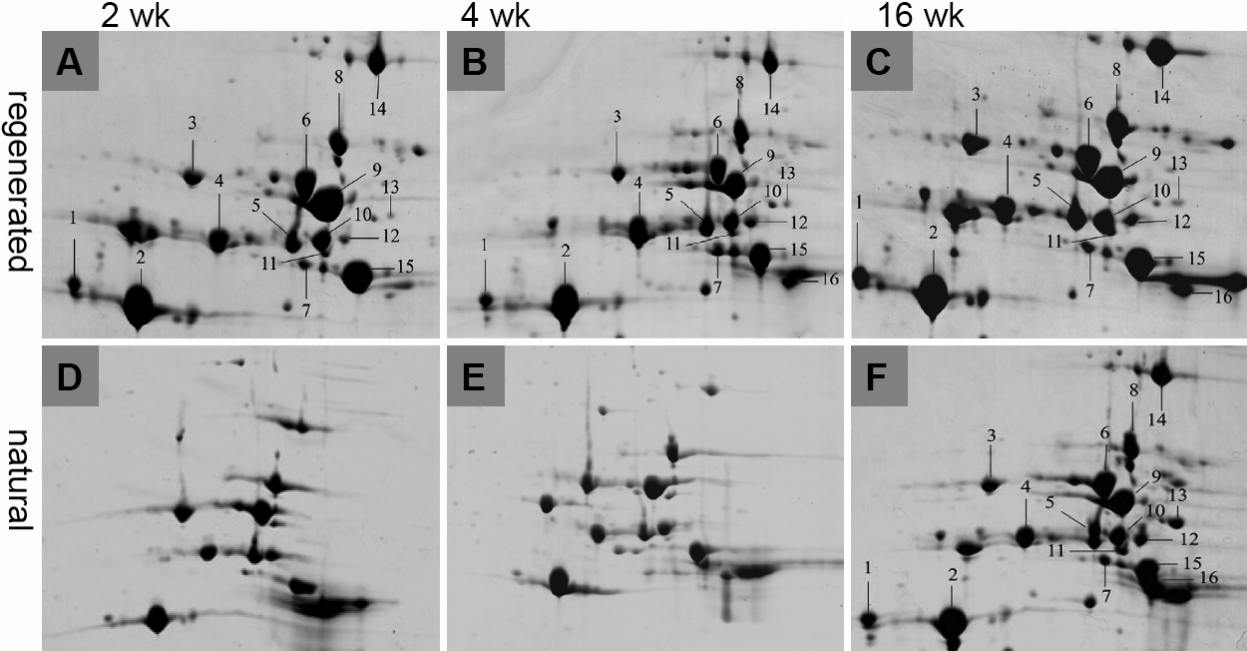
Two-dimensional electrophoresis photography of regenerated lens. **A**-**C**: Patterns of protein spots in regenerated lenses (two weeks, four weeks, and 16 weeks). **D**-**F**: Patterns of protein spots of natural lenses (two weeks, four weeks, and 16 weeks). The protein patterns are very similar among all stages of regeneration (two weeks, four weeks, and 16 weeks) and shared a high degree of analogy with those of 16-week-old natural lenses (**F**). However, these patterns were significantly different from those of two-week-old or four-week-old natural lenses (**D**,**E**).

The protein expression profile of regenerated and control lenses was analyzed by 2-DE and mass spectrometry. Abundant protein spots detected at the 20–43 kDa range (pH 5–9) appeared likely to represent crystallins. Peptide mass fingerprinting (PMF) of 16 of these spots demonstrated that 14 of them were indeed crystallins (Appendix 1).The patterns of protein spots were very similar among all the stages of regenerated lenses (two weeks, four weeks, and 16 weeks; Figure 5A-C) and shared a high degree of analogy with those of 16-week-old natural lenses (Figure 5F). However, these patterns were significantly different from those of two-week-old and four-week–old natural lenses (Figure 5D,E). Specifically, the patterns of two-week-old regenerated lenses showed remarkable analogy with those of the16-week-old natural lens (Figure 5F) but not with two-week-old natural control lenses (Figure 5D). As expected, there was not a close match between the regenerating stage of the lens and the growth stage of the normally developing rabbit.

Although the patterns of regenerated 16-week-old lenses and 16-week-old control lenses were very similar, the protein profiles of 16-week-old regenerated lenses revealed relatively higher intensity of spot 2 (αA), spot 8 (βB1), spot 9 (βB2), and spot 10 (βA2) and relatively lower intensity of spot 12 (βA3) and spot 13 (βB3) when compared to 16-week-old natural lenses using computer analysis (Table 1). The same magnitude changes in the intensity of some spots corresponding to several crystallins were also observed among 2-, 4-, and 16-week-old regenerated lenses.

**Table 1:** Changes in normalized spot volume of crystallin subunits in regenerated rabbit lens.

**Spot No.**	**Crystallin name**	**Reg1**	**Reg2**	**Reg3**	**Control**
1	/*	2.12	3.32	1.89	2.11
2	αA	11.41	12.31	17.47	10.15
3	/*	3.43	2.02	4.52	3.41
4	βA4	7.82	6.32	5.34	4.52
5	βA3 (contains: βA1)	5.16	4.39	5.72	5.65
6	βB3	5.81	5.8	6.13	5.56
7	αB	2.02	1.12	1.22	1.45
8	βB1	5.53	4.05	7.33	4.57
9	βB2	14.58	10.89	13.63	9.36
10	βA2	4.78	5.32	7.03	5.03
11	βA3	1.40	1.43	1.28	1.34
12	β A3	0.77	1.67	1.16	1.88
13	βB3	0.13	0.19	0.09	1.74
14	λ	5.27	6.43	5.39	6.41
15	αB	14.77	12.11	14.16	13.82
16	γC	/	1.43	1.28	1.68

Normalized spot volume of each crystallin subunit is an average of three or four gels of each group. A spot is calculated by dividing its volume by the total volume and multiply by 100. Reg1: lenses from a two-week-old regenerated lens; Reg2: lenses from a four-week-old regenerated lens, Reg3: lenses from a 16-week-old regenerated lens; Control: natural lenses from a 16-week-old rabbit. The asterisk indicates that the protein spot was not identified as crystalline.

## Discussion

Gwon and coworkers [11-14,28] have extensively studied rabbit lens regeneration, observing the regenerative process in the New Zealand albino rabbit after endocapsular lens extraction. They studied the histological changes and protein composition of regenerated lenses, showing that regenerated rabbit lenses differentiate normally at the equatorial zone and produce α-, β-, and γ-crystallins in proportions similar to those of natural lenses [11,12]. Based on these observations, they proposed that lens regeneration mirrors the stages seen in embryonic development.

We followed Gwon’s methods to establish the rabbit lens regeneration models. Relatively clear regenerated lenses but with a nuclear opacity and some opaque spots in the cortex were formed within 12–16 weeks after surgery. Similar to what was reported by Gwon et al. [11,13], we found by light microscopy that remnant lens epithelial cells differentiate at the lens equator and new lens fibers aligned in a concentric pattern, which is histologically similar to that of the early stage of lens development. However, TEM revealed lens equatorial epithelial cells with morphological changes including overly dense, indented nuclei, edematous mitochondria, and expanding endoplasmic reticulum. Although more complete analysis will be needed to draw definitive conclusions, these results suggest changes usually associated with cellular senescence and not with the normal lens developmental process.

More importantly, proteomic 2-DE with peptide mass fingerprinting identification showed that although the regenerated lens indeed contains all the major α-, β-, γ-crystallins, there was not a close match between the protein expression pattern of the lens substance in the regenerating stage of lens and the pattern in the growth stage of the normally developing lens.

The rabbits selected for surgery were about 12 weeks old. We began to count the lens regeneration time when the surgery was performed. For example, two weeks after surgery, we recorded a regenerated lens as two weeks old, although the rabbit’s age was 14 weeks. Four weeks after surgery, the rabbit age is actually 16 weeks, but we recorded the regenerated lens as four weeks, and so on. We compared 2-, 4-, and 16-week-old regenerated lens substances with natural lens substances from rabbits aged two weeks, four weeks, and 16 weeks. 2-DE showed that the protein spots of all regenerated lenses (two weeks, four weeks, and 16 weeks) were remarkably analogous to those of natural clear lenses from adult 16-week-old rabbits. The similarity was particularly evident in the major crystallin subunit fractions. Interestingly, however, these fractions were not at all analogous to natural lenses of earlier developing age rabbits, particularly when the two-week-old natural lenses were compared with the two-week-old regenerated lenses.

During the development of the natural lens, the protein expression pattern is in a continuous changing process. However, there is a more noticeable level of change from the newborn stage or very early developing stage to adult stages [29-32]. Although we did not follow the changes in the protein expression over time in the regenerated lens, the protein expression pattern of the early regeneration stage (such as two-week-old regenerated lens) showed clear analogy with those of adult rabbit natural lens (16 weeks old) with respect to crystallin expression. The protein patterns are very similar among all the stages of regeneration (two weeks, four weeks, and 16 wks) from early regeneration time to late regeneration time. The reason could be that even for the early regenerating lens, the rabbit’s actual age is mature enough. Therefore, the new regenerating lens substance has the similar protein pattern with the natural adult lens but such a dissimilar protein pattern from newborn or early developing lens. Moreover, although 2-D gels show a similarity in protein expression pattern between 16-week-old regenerated lenses (rabbit age 28 wks) and 16-week-old natural lenses (rabbit age 16 weeks), further quantitative comparison revealed that the amounts of crystallin subunit spots in 16-week-old regenerated lens materials (rabbit age 28 weeks) were different from those found in the natural lens materials of 16-week-old rabbits. However, the real meaning of the differences will require further investigation because the similar magnitude of the increases and decreases of some crystalline subunit spots were also observed among 2-, 4-, and 16-week-old regenerated lenses.

In summary, rabbit lens regeneration is the result of pre-equatorial epithelial cell proliferation and differentiation, regardless TEM showed the morphological changes of the nuclei and organelles in lens epithelial cells, consistent with those previously reported that have lead to the hypothesis that lens regeneration mimics the process of lens development. However, proteomic analysis revealed that the protein profile of regenerated lenses (even two-week-old regenerated lenses) was not analogous to the one existing in the natural lenses in the early developing stage (two-week-old rabbits) but shares a much clearer similarity with profile of the natural lenses of adult rabbits (16 weeks old). This finding suggests that the regrowth of lens materials in the lens capsule after endocapsular phacoemulsification might actually represent the regeneration of “mature” lens substances. Our results have led us to the conclusion that the regeneration process does not exactly mimic embryogenesis. More studies are needed to understand the synthesis of lens crystallin proteins as well as posttranslational modification changes in the regenerative process. Such understanding is critical for tissue engineering efforts aimed at regenerating fully functional clear lenses rather than old cataractous lenses.

## Appendix 1. Rabbit crystallins subunits identified by peptide mass fingerprinting.

To access the data, click or select the words “Appendix 1.” This will initiate the download of a compressed (pdf) archive that contains the file.
